# A Fully-Automated Subcortical and Ventricular Shape Generation Pipeline Preserving Smoothness and Anatomical Topology

**DOI:** 10.3389/fnins.2018.00321

**Published:** 2018-05-17

**Authors:** Xiaoying Tang, Yuan Luo, Zhibin Chen, Nianwei Huang, Hans J. Johnson, Jane S. Paulsen, Michael I. Miller

**Affiliations:** ^1^Sun Yat-sen University-Carnegie Mellon University Joint Institute of Engineering, Sun Yat-sen University, Guangzhou, China; ^2^Sun Yat-sen University-Carnegie Mellon University Shunde International Joint Research Institute, Shunde, China; ^3^School of Electronics and Information Technology, Sun Yat-sen University, Guangzhou, China; ^4^Department of Electrical and Computer Engineering, Carnegie Mellon University, Pittsburgh, PA, United States; ^5^Department of Psychiatry, University of Iowa Carver College of Medicine, Iowa City, IA, United States; ^6^Center for Imaging Science, Johns Hopkins University, Baltimore, MD, United States; ^7^Institute for Computational Medicine, Johns Hopkins University, Baltimore, MD, United States; ^8^Department of Biomedical Engineering, Johns Hopkins University, Baltimore, MD, United States

**Keywords:** subcortical structures, lateral ventricle, shape, surface filtering, large deformation diffeomorphic metric mapping, surface triangularization

## Abstract

In this paper, we present a fully-automated subcortical and ventricular shape generation pipeline that acts on structural magnetic resonance images (MRIs) of the human brain. Principally, the proposed pipeline consists of three steps: (1) automated structure segmentation using the diffeomorphic multi-atlas likelihood-fusion algorithm; (2) study-specific shape template creation based on the Delaunay triangulation; (3) deformation-based shape filtering using the large deformation diffeomorphic metric mapping for surfaces. The proposed pipeline is shown to provide high accuracy, sufficient smoothness, and accurate anatomical topology. Two datasets focused upon Huntington's disease (HD) were used for evaluating the performance of the proposed pipeline. The first of these contains a total of 16 MRI scans, each with a gold standard available, on which the proposed pipeline's outputs were observed to be highly accurate and smooth when compared with the gold standard. Visual examinations and outlier analyses on the second dataset, which contains a total of 1,445 MRI scans, revealed 100% success rates for the putamen, the thalamus, the globus pallidus, the amygdala, and the lateral ventricle in both hemispheres and rates no smaller than 97% for the bilateral hippocampus and caudate. Another independent dataset, consisting of 15 atlas images and 20 testing images, was also used to quantitatively evaluate the proposed pipeline, with high accuracy having been obtained. In short, the proposed pipeline is herein demonstrated to be effective, both quantitatively and qualitatively, using a large collection of MRI scans.

## Introduction

Analyzing the shape of subcortical and ventricular structures subjected to brain disorders is an area of ever growing importance, especially in the fields of neurodegenerative diseases such as Alzheimer's disease (Qiu et al., 2009b; Wang et al., 2011; Shi et al., 2013, 2015; Tang et al., 2014, 2015b; Miller et al., 2015), Huntington's disease (HD) (van den Bogaard et al., 2011; Younes et al., 2014; Faria et al., 2016), and Parkinson's disease (Sterling et al., 2013; Nemmi et al., 2015) as well as various neurodevelopmental disorders (Knickmeyer et al., 2008; Rimol et al., 2010; Seymour et al., 2017). The anatomical shapes of the structures of interest in those cases are usually represented using a mesh that can be created from the corresponding structural volumetric segmentation. In more detail, generating a segmentation-based shape representation of a specific structure of interest (such as the left hippocampus) consists of two steps: (1) segmenting that structure of interest from a structural magnetic resonance image (MRI), resulting in a 3D volumetric segmentation; (2) converting that volumetric segmentation into a smooth surface representing the structural segmentation's boundary (Levine et al., 2012).

The fully automated segmentation of subcortical and ventricular structures, based on structural MRIs, is a well-established field of research, with a variety of highly accurate algorithms having already been developed (Barra and Boire, 2001; Khan et al., 2008; Powell et al., 2008; Patenaude et al., 2011; Chakravarty et al., 2013; Tang et al., 2015c). As for the generation of surfaces, image-based meshing is typically employed, especially when creating computer models for computational fluid dynamics and finite element analysis (Young et al., 2008; Chen et al., 2013; Chernikov et al., 2013; Foteinos and Chrisochoides, 2013; Zhang, 2013). More recently, segmentation based meshing has also been applied to the medical imaging field, see Zhang (2013) for a general introduction. One of the most representative meshing techniques is the marching cubes algorithm, which has been incorporated into a number of commercial and non-commercial software packages. The marching cubes algorithm takes a 3D segmentation image as its input and outputs surface data in the form of a triangulated mesh, represented using vertices and faces.

Combining what we have just outlined leads to an “automated volume segmentation + marching cubes based surface generation” pipeline for subcortical and ventricular structures. Such a procedure may well be vulnerable to noise induced by inaccurate segmentations, resulting in disconnected regions or holes within the surface (Qiu and Miller, 2008). In addition, it is plausible that the marching cubes algorithm is liable to miss thin subregions of a structure of interest such as the thin “bridge” connecting the inferior horn and the main body of the lateral ventricle (Qiu and Miller, 2008). In other words, the resulting surface may not have the correct anatomical topology. Furthermore, even for a structure of interest with a highly accurate segmentation and an “easy” topology (a relatively simple shape), it is likely that the marching cubes algorithm will not deliver surfaces of a sufficient smoothness. Indeed, it is a most challenging task to extract the structure of interest's surface with high accuracy, correct anatomical topology, and sufficient smoothness in the same instance. To ensure a high degree of accuracy in the surface, a precise volumetric segmentation and a high fidelity in the surface with respect to the corresponding volumetric segmentation is required. Naturally, to ensure a correct anatomical topology, a surface generation approach that is devised around the notion of preserving the anatomical topology of the structure of interest is needed. Meanwhile, the classic filtering and smoothing approaches may not be sufficient to ensure the required smoothness without sacrificing the fidelity to the corresponding volumetric segmentation.

Alternatives to the aforementioned combination are certainly possible and there are numerous existing pipelines that can generate smooth subcortical structural shapes directly from MRIs. In contrast to a binary segmentation procedure for shape generation, those pipelines generally employ shape modeling for their segmentation purposes (Heimann and Meinzer, 2009; Patenaude et al., 2011). In other words, the structural shapes were not created from the binary segmentation, but directly from the dense MR images. The main limitation of these shape-modeling based approaches is the lack of flexibility in relation to individual components; one may desire the ability to utilize a more accurate segmentation algorithm or a more sophisticated meshing algorithm.

It is in the context of all of the above that we propose a fully-automated subcortical and ventricular shape generation pipeline which satisfies the demand for accuracy (both topological and otherwise) and smoothness in four steps: (1) automatically segment the subcortical and ventricular structures of interest using the raw structural MRI data acquired from a scanner; (2) create a study-specific template shape with the correct anatomical topology and sufficient surface smoothness; (3) create a triangulated mesh from each binary segmentation obtained in step (1) using the marching cubes algorithm; (4) filter and smooth the surfaces generated in step (3) in a deformation based manner.

To perform the initial segmentation, we employ a fully-automated segmentation pipeline, the diffeomorphic multi-atlas likelihood fusion (MALF) algorithm (Tang et al., 2013), the accuracy of which in segmenting subcortical and ventricular structures has been validated on a variety of MRI datasets (Tang et al., 2015c). Instead of applying the marching cubes algorithm directly, to generate a corresponding triangulated mesh from the segmentation of MALF with the desired properties, we rely on deformation based shape generation in the setting of large deformation diffeomorphic metric mapping (LDDMM) for surfaces (Vaillant and Glaunès, 2005). Given a pre-defined triangulated surface of a specific structure of interest, LDDMM is capable of preserving the topology and smoothness of that surface when registering it to a target surface. In other words, if we register a template surface with the correct anatomical topology and a high degree of smoothness to a target surface using LDDMM, the deformed surface is guaranteed to inherit that topology and smoothness from the template while being as similar as possible to the target surface. This is essentially due to the properties of diffeomorphic transformations and the capability of LDDMM to deliver the accurate diffeomorphisms needed for surface registration (Vaillant and Glaunès, 2005).

In this paper, we will first detail each of the above steps in the proposed pipeline. We then proceed to evaluate the proposed pipeline quantitatively and qualitatively using three MRI datasets. There are 16 structural MRIs in the first dataset, for each of which we manually segmented the subcortical and ventricular structures, with a view to quantitatively evaluating the performance of the proposed pipeline by comparison with the gold standard. Within the second dataset, there are a total of 1,445 structural MRIs, on which we qualitatively examine the surfaces delivered by the proposed pipeline. For the third dataset, there are 15 atlas structural MRIs and 20 testing structural MRIs, with the structures of interest being the subcortical structures that have been manually delineated. We also compared our results with those from a well-established pipeline that outputs smooth subcortical surfaces directly from dense MRIs, namely the FSL-FIRST pipeline (Patenaude et al., 2011). Three aspects were examined; the accuracy based on quantitative evaluation, the anatomy topology based on visual examination, and the smoothness based on quantitative assessment.

## Materials and methods

### PREDICT-HD

The first two datasets that feature in this work are both part of the PREDICT-HD study (https://www.predict-hd.net/) where all enrolled subjects were at risk of HD and had previously undergone elective predictive genetic testing. Subjects labeled as premanifest HD (pre-HD) are those who were found to be “gene expanded,” possessing a cytosine–adenine–guanine (CAG) ≥ 36 but not exhibiting the motor criteria consistent with a diagnosis of HD (The Huntington's Disease Collaborative Research Group, 1993). A control group was defined as subjects who were deemed “non-gene expanded,” possessing a CAG ≤ 30. Participants of PREIDCT-HD were recruited from 32 sites across the United States, Canada, Europe, and Australia and underwent longitudinal study visits consisting of a neurological motor examination, cognitive assessment, brain MRI, psychiatric and functional assessment, and blood testing for genetic and biochemical analyses. Informed written consent was obtained from all subjects before participating in this study.

Subjects with pre-HD were further divided into three subgroups (“low-HD,” “mid-HD,” and “high-HD”) based on their CAP scores, a function of their CAG repeat length and current age given by CAP = (age at study entry) × (CAG – 33.66) (Zhang et al., 2011). The three subgroups are defined according to CAP < 290 (the low-HD group), 290 ≤ CAP ≤ 368 (the mid-HD group), and CAP > 368 (the high-HD group).

### Subjects

In the first dataset, there are a total of 16 subjects (3 males and 13 females, mean age = 42.1 ± 10.1 years), including 6 control subjects, 4 low-HD subjects, 3 mid-HD subjects, and 3 high-HD subjects. Only one scan of each subject was selected, resulting in a total of 16 MRI scans in the first dataset.

For the second dataset, there are a total of 169 control subjects, including 106 females (mean age at baseline = 48.3 ± 11.2 years) and 63 males (mean age at baseline = 48.6 ± 14.8 years). Within the control group, 59 subjects had only 1 scan, 43 subjects had 2 scans, 27 subjects had 3 scans, 16 subjects had 4 scans, 15 subjects had 5 scans, 7 subjects had 6 scans, and 1 subject had 7 scans, resulting in a total of 414 MRI scans, with the average interval between two consecutive scans being 1.1 years. Within the low-HD group, there are a total of 113 subjects, including 85 females (mean age at baseline = 33.1 ± 9.1 years) and 28 males (mean age at baseline = 35.7 ± 10.8 years). In the low-HD group, 52 subjects had only 1 scan, 35 subjects had 2 scans, 12 subjects had 3 scans, 8 subjects had 4 scans, 3 subjects had 5 scans, 2 subjects had 6 scans, and 1 subject had 8 scans, resulting in a total of 225 MRI scans, with the average interval between two consecutive scans being 0.8 years. Within the mid-HD group, there are a total of 141 subjects, including 98 females (mean age at baseline = 42.1 ± 10.2 years) and 43 males (mean age at baseline = 42.4 ± 11.2 years). In the mid-HD group, 62 subjects had only 1 scan, 36 subjects had 2 scans, 14 subjects had 3 scans, 17 subjects had 4 scans, 5 subjects had 5 scans, 6 subjects had 6 scans, and 1 subject had 7 scans, resulting in a total of 312 MRI scans, with the average interval between two consecutive scans being 0.8 years. Within the high-HD group, there are a total of 227 subjects, including 136 females (mean age at baseline = 49.3 ± 10.9 years) and 91 males (mean age at baseline = 50.0 ± 11.1 years). In the high-HD group, 99 subjects had only 1 scan, 68 subjects had 2 scans, 26 subjects had 3 scans, 17 subjects had 4 scans, 8 subjects had 5 scans, 8 subjects had 6 scans, and 1 subject had 8 scans, resulting in a total of 477 MRI scans, with the average interval between two consecutive scans being 0.9 years. There are another 4 females (mean age at baseline = 44.6 ± 9.9 years) that were not identified as belonging to any group. Among those 4 subjects, 3 had been scanned once while the remainder had been scanned twice, resulting in a total of 5 MRI scans. There are another 12 MRI scans for which we could not identify their demographic and clinical information. However, given that the goal of this paper is to evaluate a surface generation pipeline rather than to compare groups of different clinical states, we retained all of the 1,445 scans from the second dataset for pipeline validation. A summary of this dataset is tabulated in Table 1.

**Table 1:** A summary of the second dataset, consisting of 1,445 MRI scans.

	**Control**		**Low-HD**	
	Male (no = 63)	Female (no = 106)	Male (no = 28)	Female (no = 85)
Baseline age	48.6 ± 14.8 years	48.3 ± 11.2 years	35.7 ± 10.8 years	33.1 ± 9.1 years
No. of scans = 1	59		52	
No. of scans = 2	43		35	
no. of scans = 3	27		12	
No. of scans = 4	16		8	
No. of scans = 5	15		3	
No. of scans = 6	7		2	
No. of scans = 7	1		0	
No. of scans = 8	0		1	
Average inter-scan interval	1.1 years		0.8 years	
	**Mid-HD**		**High-HD**	
	Male (no = 43)	Female (no = 98)	Male (no = 91)	Female (no = 136)
Baseline age	42.4 ± 11.2 years	42.1 ± 10.2 years	50.0 ± 11.1 years	49.3 ± 10.9 years
No. of scans = 1	62		99	
No. of scans = 2	36		68	
No. of scans = 3	14		26	
No. of scans = 4	17		17	
No. of scans = 5	5		8	
No. of scans = 6	6		8	
No. of scans = 7	1		0	
No. of scans = 8	0		1	
Average inter-scan interval	0.8 years		0.9 years	

High resolution anatomical MR images of the first two datasets were used in this study. Given that the PREDICT-HD study was both multi-centered and longitudinal in nature, the image acquisition procedures were heterogeneous, including multiple vendors (GE, Phillips, and Siemens), different field strengths (1.5 Tesla and 3 Tesla), and more than 20 different MR acquisition protocols (due to issues with transmission and receiver hardware). Detailed scanning information for each of the 1,445 MR scans can be found in the Supplementary Material 1.

The third dataset used in this study includes 35 brain MRI scans from the OASIS project. The manual segmentations of these images were produced by Neuromorphometrics, Inc. (http://Neuromorphometrics.com/) using the brainCOLOR labeling protocol. The data were applied in the 2012 MICCAI Multi-Atlas Labeling Challenge and are publicly accessible (https://masi.vuse.vanderbilt.edu/workshop2012/index.php/Main_Page). In the challenge, 15 subjects were used as atlases and the remaining 20 images were used for testing. For this dataset, our structures of interest are the 12 subcortical regions.

### Automated structure segmentation

**Figure 1:**
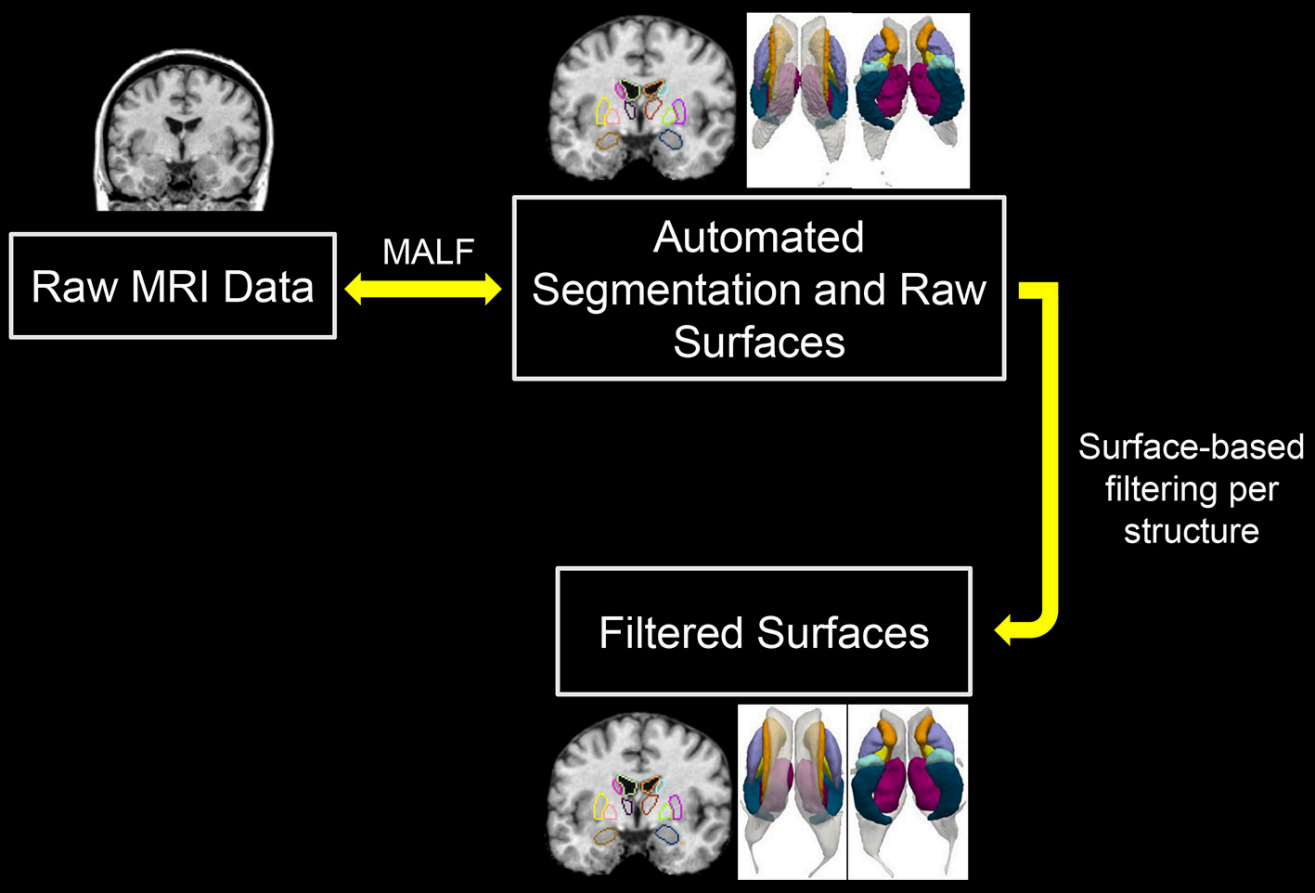
Demonstration of the workflow of the proposed pipeline. MALF, multi-atlas likelihood fusion.

As shown in Figure 1 (the work flow of the proposed pipeline), one can view this pipeline as having two major components; automated structure segmentation and surface filtering. The subcortical and ventricular structures, in both hemispheres, were extracted from each T1-weighted image using a fully-automated structure segmentation pipeline (Tang et al., 2015c) itself consisting of two steps, skull-stripping and brain structure segmentation. The underlying theoretical basis of this approach is multi-atlas likelihood-fusion (MALF) in the framework of a random deformable template model (Tang et al., 2013). This segmentation pipeline has been tested and validated on a number of datasets with relevance to various brain structures, particularly the subcortical and ventricular structures (Liang et al., 2015; Tang et al., 2015a).

In this study, the 16 T1-weighted images of the first dataset served as the atlases used in MALF to perform the automated structure segmentation for the first and the second datasets. Each structure of interest, such as the left hippocampus, was manually delineated in all 16 atlases by a team of neuroanatomists at Johns Hopkins University with more than 15 years' experience in manually tracing subcortical structures. Various sets of subcortical and ventricular atlases, used in our other studies, were all created by the same team and have proven their reliability (Tang et al., 2013, 2015c, 2016; Seymour et al., 2017). Intra- and inter-rater reliability of manual delineations by this team have been quantified in earlier studies; intra-class correlation (ICC) statistics revealed high rates of intra- and interrater reliability (intra-rater ICC ranges between 0.96 and 0.98; inter-rater ICC ranges between 0.9 and 0.93) (Qiu et al., 2009a).

To evaluate the proposed pipeline's handling of the first dataset, we adopted a leave-one-out strategy; one atlas image was treated as the to-be-segmented image while the remainder served as the atlas set used in segmenting that excluded image. When evaluating the second dataset, we continued to use these 16 atlases for segmentation via MALF. For the third dataset, the 15 atlas images were used to segment the subcortical structures in each of the 20 testing images.

### Surface generation

With the binary segmentation of the structures of interest completed using the structure segmentation procedure discussed above, we proceeded to create a triangulated mesh contouring the boundary of the segmentation using the marching cubes algorithm. The marching cubes algorithm yields triangulated surfaces with a high fidelity to the segmentation. Thus, when the segmentation is lacking accuracy, the marching cubes algorithm will be incapable of correcting the mistakes incurred during the segmentation step. In addition, the resulting surface may well be insufficiently smooth for our purposes. To overcome these limitations, one potential approach is to register a template surface to a target surface (the raw structure surface created from the marching cubes algorithm). The template surface is supposed to have correct anatomical topology and sufficient smoothness. The deformed template surfaces are therefore expected to have geometric characteristics identical to those of the target surfaces while possessing the topology and connectivity of the template surface.

In our pipeline, the template surface came from one of the 16 subjects in the first dataset. The 14 structures of interest for the selected subject were manually delineated with care taken to ensure both segmentation accuracy and boundary smoothness during the manual delineation. That specific subject was chosen based on three considerations: (1) the area of the subject's surface should be close to the mean area across all 16 surfaces from the manual segmentations; (2) the geometry and topology of the subject's surface should be correct based on visual examination; (3) the selected surface should be sufficiently smooth quantitatively and qualitatively.

In creating the template surface, instead of using the marching cubes algorithm, we adopted the Delaunay algorithm for triangulation (Lee and Schachter, 1980; Shewchuk, 2002) to guarantee further smoothness. We have noticed, however, that the Delaunay algorithm is much less stable than that of the marching cubes, even though it yields smoother results. This is our rationale for using marching cubes for the triangulation of the raw structure surfaces rather than the Delaunay algorithm.

With the template surface and target surfaces for each structure of interest created, we performed a rigid alignment of the surfaces and then proceeded to the LDDMM surface registration (Vaillant and Glaunès, 2005). Specifically, the template surface was rigidly aligned (rotation and translation) to the target surface, with the optimal rigid transformation between the vertex sets of the two surfaces obtained by minimizing a score that combines registration and soft assignment. After that, the LDDMM surface registration was performed from the rigidly aligned template surface to the target surface. Details on the “rigid + LDDMM” surface registration pipeline can be found in our previous work (Tang et al., 2014). After obtaining all of the rigid and diffeomorphic transformations between the template surface and the target surfaces, we applied these transformations in turn to the template surface, generating a deformed template surface for each structure of interest in each subject MRI. This deformed template surface is the result of our proposed pipeline, a smooth surface of a subcortical and ventricular structure of interest in an individual MRI scan.

### Evaluation criteria

As we have the gold standard—manual segmentations—at our disposal for the first and the third datasets, we quantitatively computed the accuracy and reliability of the proposed pipeline through the use of the following evaluation metrics:

- Dice similarity coefficient (DSC)
  (1) $$\begin{array}{r} DSC\left(A,B\right)=2\frac{V\left(A\cap B\right)}{V\left(A\right)+V\left(B\right)} \end{array}$$

where *V*(*A*) and *V*(*B*) are the volumetric measurements of segmented images *A* and *B*. For example, *A* may represent the binary segmentation of the left hippocampus from manual delineation while *B* represents the corresponding automated segmentation from MALF.

- Absolute volume difference (AVD)
  (2) $$\begin{array}{r} AVD\left(A,B\right)=\frac{\left|V\left(A\right)-V\left(B\right)\right|}{\left(V\left(A\right)+V\left(B\right)\right)/2} \end{array}$$

where *V*(*A*) and *V*(*B*) are again the volumetric measurements of segmented images *A* and *B*.

- Correlation coefficient

For the third quantitative comparison metric, we employed the Pearson product-moment correlation coefficient (PCC) between the volumetric measurements of the two segmentations in comparison, for example those of the manual segmentation and the MALF-derived automated segmentation.

In addition to evaluating the segmentation accuracy using the first and the third datasets, we also assessed the smoothness of the resulting surfaces quantitatively and qualitatively (through visual examination by several raters) using all three datasets. The smoothness of a surface was quantified using the following metric:

- Geometric Laplacian (GL)
  (3) $$\begin{array}{r} GL\left(v\right)=v-\frac{\sum_{i\in n\left(v\right)}l_{i}^{-1}v_{i}}{\sum_{i\in n\left(v\right)}l_{i}^{-1}} \end{array}$$

where *n*(*v*) is the index set of the vertices *v*_*i*_ which are themselves the direct neighbors of *v*, and *l*_*i*_ is the Euclidean distance from *v* to *v*_*i*_. *GL*(*v*) represents a kind of measure of roughness: the higher it is, the rougher is the surface around *v*. The GL of a surface is computed as the sum of the norm of all vertex-wise GL vectors, namely $GL=\underset{v}{\sum }{\left||GL\left(v\right)|\right|}_{2}$.

### Group comparisons

In our first experiment, we compared results from the proposed pipeline, in terms of both volumetric segmentations and triangulated surfaces, with those before filtering (obtained from MALF) using all three datasets. Their results were also compared to the gold standard of the first and the third datasets. In the first experiment, our structures of interest included all the 14 subcortical and lateral ventricle structures for the first two datasets and the 12 subcortical structures for the third dataset. In the second experiment, we performed a comparison with a state-of-the-art pipeline, FSL-FIRST, that outputs volumetric segmentations as well as smooth triangulated surfaces of subcortical structures as well. This experiment was conducted on the first dataset and analyzed the 12 subcortical structures only, as FSL-FIRST does not output lateral ventricle results. Student's *t*-tests were employed to evaluate the significance of a group difference in all settings.

## Results

### The first experiment

In Tables 2–4, we respectively detail the mean and standard deviations of the DSCs, the AVDs, and the PCCs for each of the 14 structures of interest of the first dataset when calculated under the three possible comparisons; the raw automated segmentations from MALF vs. the manual segmentations, the raw automated segmentations from MALF vs. the filtered automated segmentations, as well as the filtered automated segmentations vs. the manual ones. The corresponding results on the 12 subcortical structures of the third dataset are demonstrated in the Supplementary Material 2 (Table S1). Please note, the filtered automated segmentations were generated from the smoothly deformed surfaces via nearest neighbor assignment. As shown in the first column of each of the three tables, the raw automated segmentations obtained from MALF are highly accurate when compared to the gold standard. This illustrates the accuracy of the first step of our surface generation pipeline. For the second step, generating a smoothed version of the raw surface, we achieved a high fidelity, as is demonstrated in the second column in each of the three tables. Comparing the final results, the filtered surface based segmentations, with the gold standard, the accuracy is again high (the third column of each of the three tables) and indeed similar to that of the raw accuracy.

**Table 2:** The average Dice overlap coefficients between every pairing of the three sets of segmentation results (manual segmentation, raw automated segmentation, and filtered automated segmentation) over the 16 MRI scans of the first group for each of the 14 subcortical and ventricle structures.

	**Manual vs. Raw Auto**	**Raw Auto vs. Filtered Auto**	**Manual vs. Filtered Auto**
Left caudate	0.914 ± 0.039	0.958 ± 0.008	0.913 ± 0.039
Right caudate	0.901 ± 0.028	0.957 ± 0.006	0.899 ± 0.027
Left pallidum	0.902 ± 0.023	0.957 ± 0.006	0.899 ± 0.024
Right pallidum	0.907 ± 0.018	0.957 ± 0.005	0.906 ± 0.022
Left putamen	0.928 ± 0.010	0.965 ± 0.005	0.928 ± 0.009
Right putamen	0.934 ± 0.012	0.966 ± 0.005	0.934 ± 0.009
Right thalamus	0.922 ± 0.011	0.969 ± 0.004	0.924 ± 0.011
Left thalamus	0.927 ± 0.009	0.970 ± 0.004	0.929 ± 0.008
Left amygdala	0.874 ± 0.017	0.943 ± 0.010	0.874 ± 0.020
Right amygdala	0.866 ± 0.025	0.946 ± 0.008	0.870 ± 0.025
Left hippocampus	0.917 ± 0.009	0.939 ± 0.006	0.909 ± 0.011
Right hippocampus	0.910 ± 0.013	0.943 ± 0.007	0.907 ± 0.014
Left ventricle	0.925 ± 0.023	0.891 ± 0.047	0.858 ± 0.058
Right ventricle	0.922 ± 0.027	0.902 ± 0.048	0.866 ± 0.059

**Table 3:** The average absolute volume differences between every pairing of the three sets of segmentation results (manual segmentation, raw automated segmentation, and filtered automated segmentation) over the 16 MRI scans of the first group for each of the 14 subcortical and ventricle structures.

	**Manual vs. Raw Auto**	**Raw Auto vs. Filtered Auto**	**Manual vs. Filtered Auto**
Left caudate	0.076 ± 0.092	0.011 ± 0.005	0.078 ± 0.088
Right caudate	0.098 ± 0.080	0.010 ± 0.005	0.100 ± 0.079
Left pallidum	0.103 ± 0.075	0.016 ± 0.007	0.108 ± 0.079
Right pallidum	0.082 ± 0.061	0.018 ± 0.006	0.086 ± 0.071
Left putamen	0.037 ± 0.027	0.007 ± 0.005	0.035 ± 0.027
Right putamen	0.055 ± 0.026	0.008 ± 0.004	0.055 ± 0.023
Right thalamus	0.090 ± 0.038	0.002 ± 0.002	0.092 ± 0.039
Left thalamus	0.075 ± 0.035	0.003 ± 0.003	0.075 ± 0.036
Left amygdala	0.082 ± 0.041	0.018 ± 0.005	0.077 ± 0.045
Right amygdala	0.069 ± 0.072	0.015 ± 0.005	0.065 ± 0.072
Left hippocampus	0.064 ± 0.029	0.014 ± 0.006	0.076 ± 0.029
Right hippocampus	0.065 ± 0.031	0.014 ± 0.005	0.074 ± 0.033
Left ventricle	0.078 ± 0.053	0.006 ± 0.005	0.080 ± 0.055
Right ventricle	0.082 ± 0.057	0.010 ± 0.006	0.088 ± 0.061

**Table 4:** The Pearson product-moment correlation coefficients between every pairing of the three sets of segmentation results (manual segmentation, raw automated segmentation, and filtered automated segmentation) over the 16 MRI scans of the first group for each of the 14 subcortical and ventricle structures.

	**Manual vs. Raw Auto**	**Raw Auto vs. Filtered Auto**	**Manual vs. Filtered Auto**
Left caudate	0.821	1.000	0.827
Right caudate	0.821	1.000	0.829
Left pallidum	0.737	0.999	0.741
Right pallidum	0.835	0.999	0.835
Left putamen	0.980	1.000	0.980
Right putamen	0.965	1.000	0.966
Right thalamus	0.899	1.000	0.898
Left thalamus	0.906	0.999	0.908
Left amygdala	0.633	0.999	0.641
Right amygdala	0.716	0.999	0.711
Left hippocampus	0.795	0.998	0.809
Right hippocampus	0.804	0.999	0.819
Left ventricle	0.996	1.000	0.996
Right ventricle	0.990	1.000	0.990

**Figure 2:**
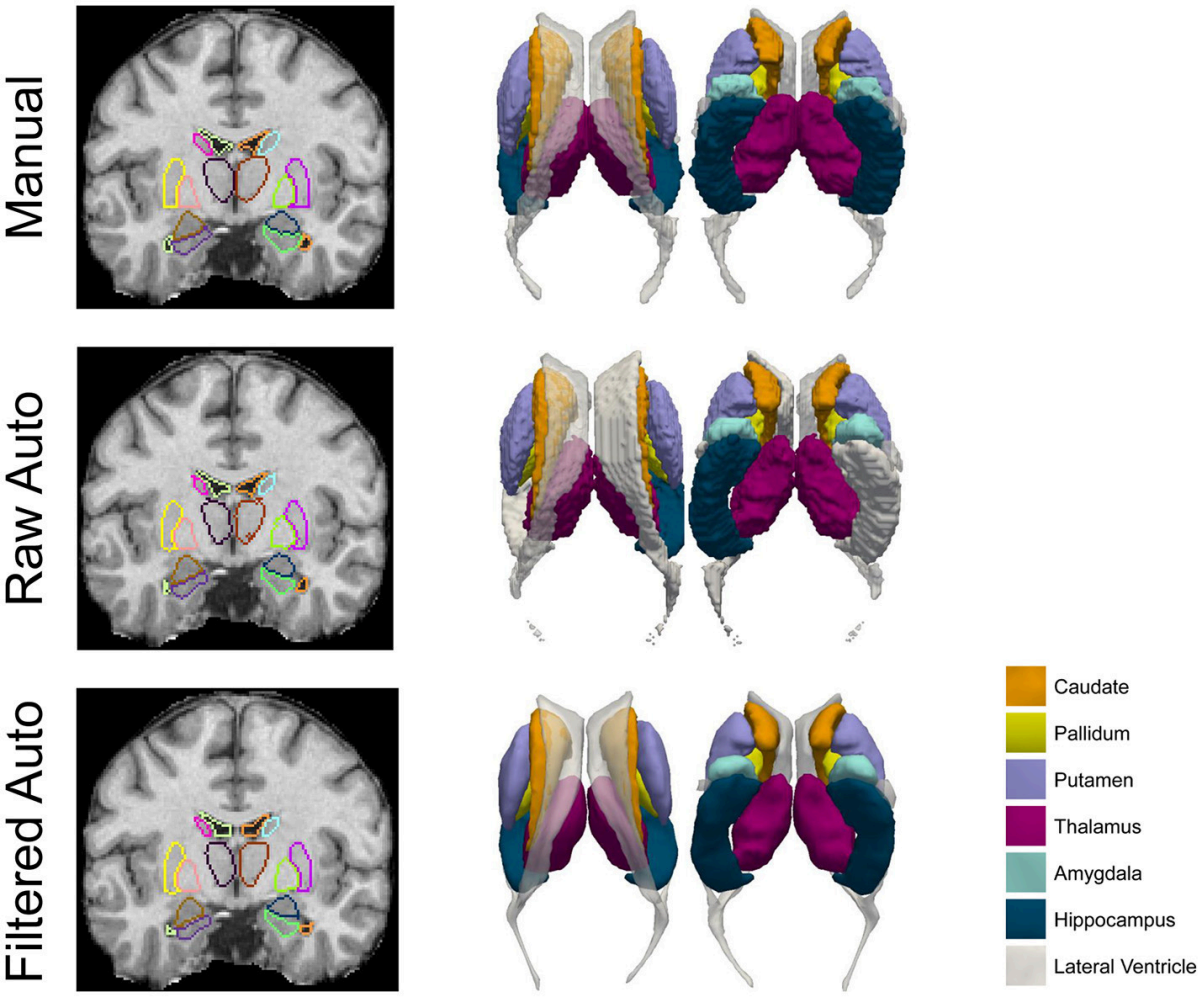
A comparison of the manual results, the raw automated segmentation results, and the filtered automated results, for the 7 subcortical and ventricular structures (both left and right) of one representative subject. Both segmentations (left column) and the corresponding triangulated surfaces (right column) are presented.

Results on comparing the smoothness of the surfaces of those three approaches for the first and the third datasets are respectively demonstrated in Table 5 and the Supplementary Material 2 (Table S2). Clearly, for each of the structures of interest, surfaces from the proposed pipeline are significantly smoother (*p* << 1E^−10^) than not only the raw automated results from MALF but also the manual results. In Figure 2, we present comparison results for the three methods (manual, raw automated, and filtered automated), in terms of segmentations that are superimposed on the structural MR image (for better visualization) and the corresponding surfaces, for one representative subject. Evidently, the proposed method is capable of capturing thin regions of a structure of interest, such as in the lateral ventricle, and thus preserving the structure's anatomical topology. Furthermore, even when compared with the gold standard surfaces created from the marching cubes algorithm, the surfaces delivered by the proposed pipeline are much smoother.

**Table 5:** Smoothness quantification, as measured by the Geometric Laplacian, of the four sets of surface results [manual, raw automated (MALF), filtered automated (proposed), and FSL-FIRST] over the 16 MRI scans of the first group for the 12 subcortical structures.

	**Manual**	**MALF**	**Proposed**	**FSL-FIRST**
Left caudate	562.371 ± 70.610	619.822 ± 61.332	192.202 ± 8.967	187.216 ± 9.973
Right caudate	553.225 ± 81.988	599.452 ± 58.889	208.535 ± 7.896	193.338 ± 12.298
Left pallidum	249.926 ± 27.753	250.861 ± 30.475	87.136 ± 3.919	73.241 ± 4.416
Right pallidum	246.651 ± 20.956	254.202 ± 28.085	78.655 ± 3.506	67.427 ± 4.129
Left putamen	509.561 ± 61.449	560.402 ± 54.812	176.756 ± 6.163	123.851 ± 8.138
Right putamen	515.220 ± 57.479	568.109 ± 48.324	181.189 ± 6.611	126.411 ± 8.534
Right thalamus	634.443 ± 45.398	724.972 ± 65.240	197.801 ± 5.349	133.038 ± 5.835
Left thalamus	626.319 ± 46.549	708.264 ± 56.974	194.041 ± 7.095	132.420 ± 5.196
Left amygdala	232.511 ± 22.514	244.034 ± 18.729	84.181 ± 1.899	195.033 ± 13.753
Right amygdala	218.056 ± 22.433	229.562 ± 21.813	77.585 ± 2.445	167.832 ± 10.335
Left hippocampus	581.167 ± 27.421	607.406 ± 37.712	183.013 ± 4.637	77.366 ± 6.519
Right hippocampus	595.273 ± 32.929	619.702 ± 45.359	181.883 ± 4.959	80.187 ± 5.993

**Figure 3:**
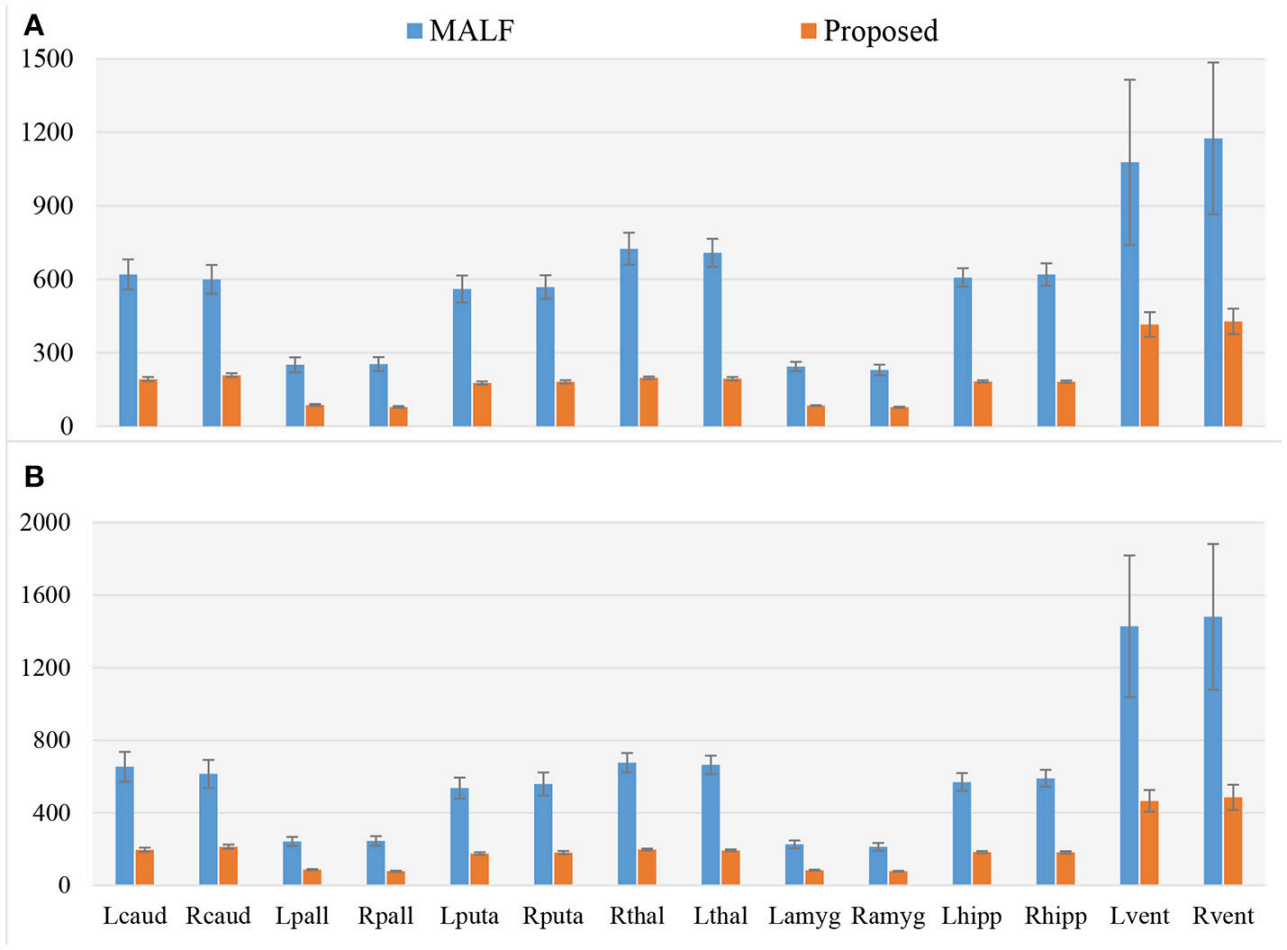
A comparison of the smoothness, as assessed by the Geometric Laplacian, of surfaces from MALF (the raw automated segmentation results) and the proposed method (the filtered automated results), for the 7 subcortical and ventricular structures (both left and right) for both datasets. Lcaud, left caudate; Rcaud, right caudate; Lpall, left pallidum; Rpall, right pallidum; Lputa, left putamen; Rputa, right putamen; Lthal, Left Thalamus; Rthal, right thalamus; Lamyg, left amygdala; Ramyg, right amygdala; Lhipp, left hippocampus; Rhipp, right hippocampus; Lvent, left ventricle; Rvent, right ventricle. **(A,B)** Respectively denote the results for the first and the second dataset.

**Figure 4:**
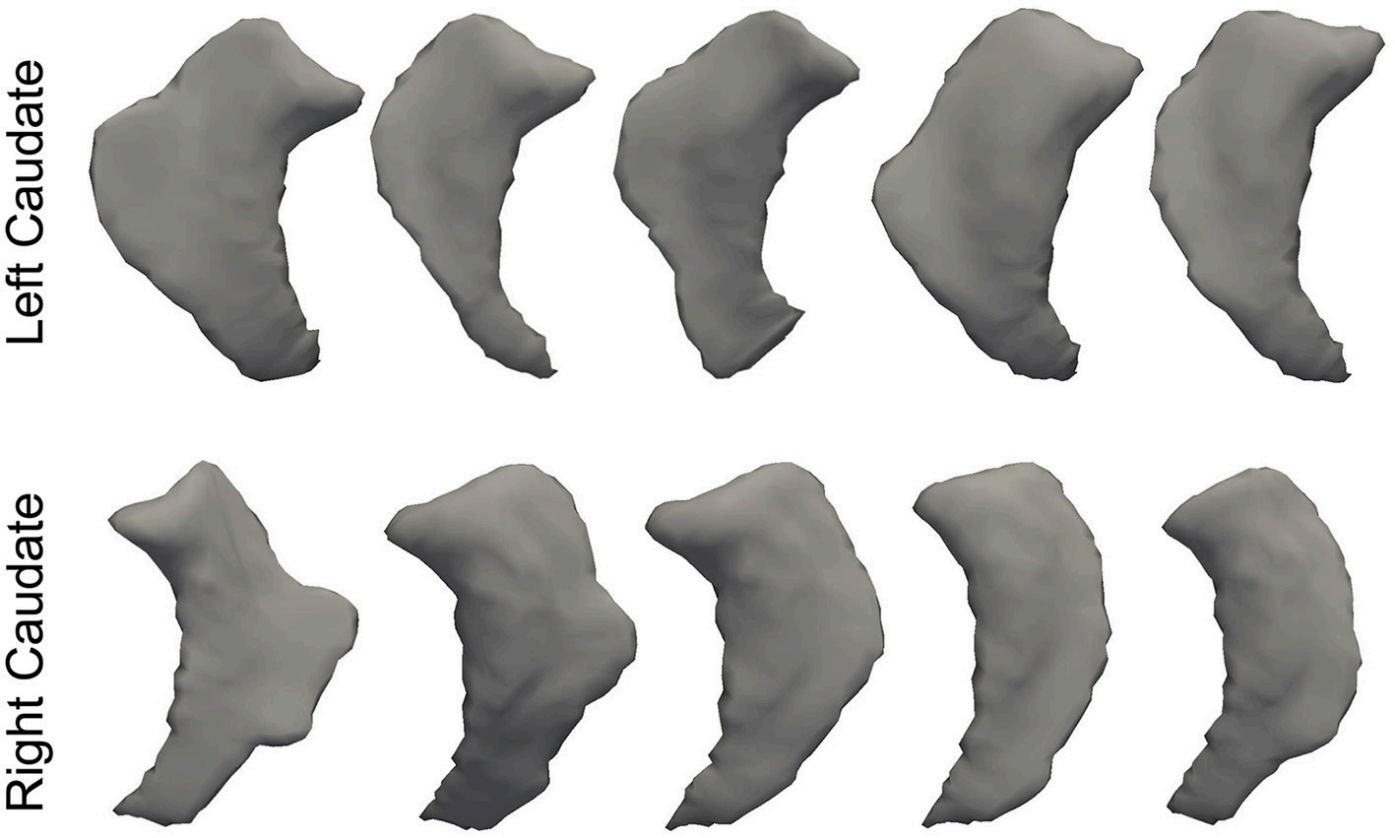
Representative failure cases for the left caudate **(top)** and the right caudate **(bottom)** from the second dataset.

**Figure 5:**
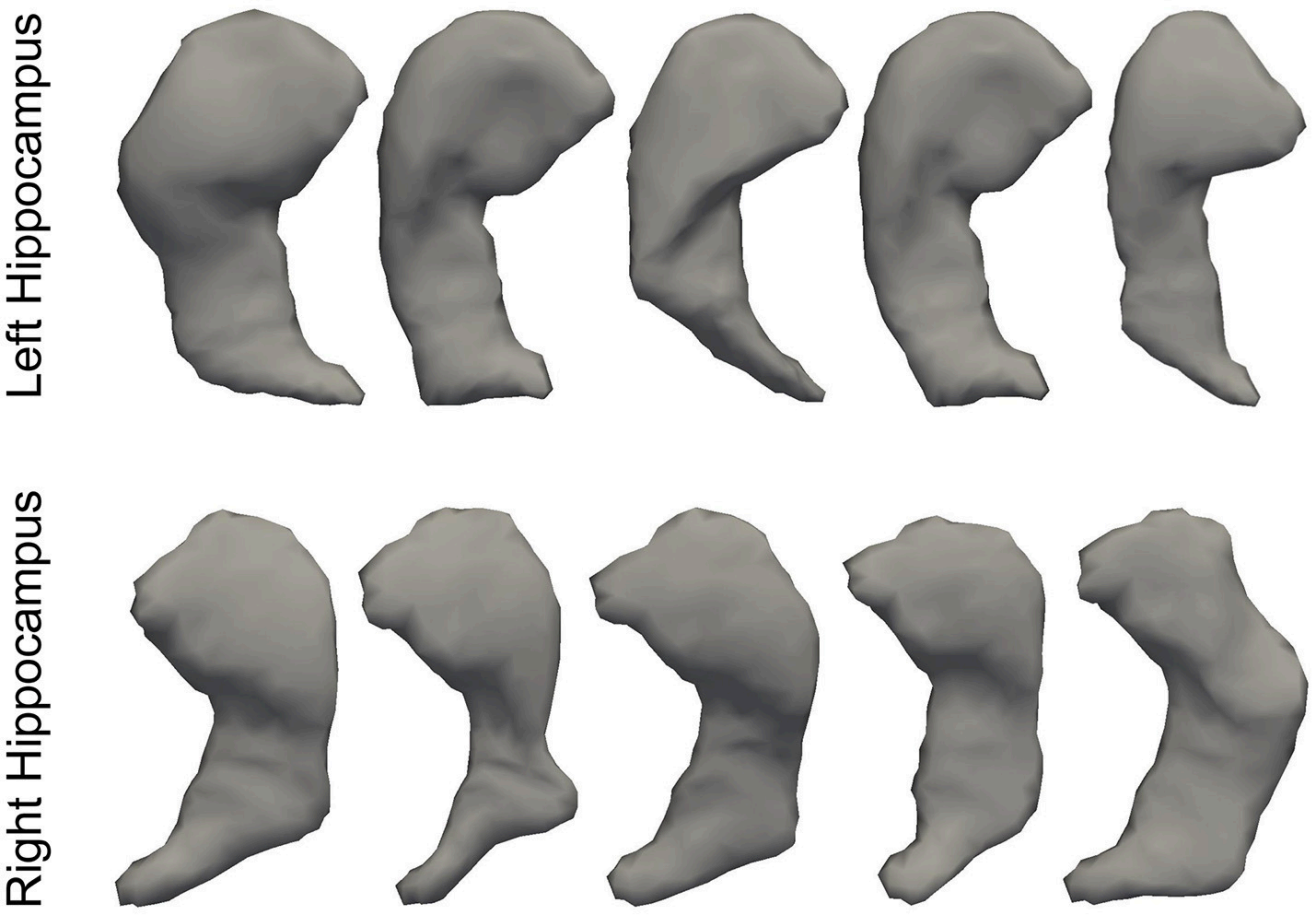
Representative failure cases for the left hippocampus **(top)** and the right hippocampus **(bottom)** from the second dataset.

In Figure 3, we illustrate the smoothness comparison results of both datasets before and after deformation based filtering, from which a significant increase in smoothness was observed for each structure in both datasets. In addition to smoothness, the segmentation accuracy of the second dataset were also visually examined independently by three experienced raters. We found that on the bilateral putamen, globus pallidus, amygdala, thalamus, and lateral ventricle, the proposed pipeline delivered sufficiently well-generated surfaces for all 1,445 scans. In other words, the failure rate for any of those 5 structures in both hemispheres is 0%. For the other subcortical structures the number of surfaces found to be flawed were as follows: 19 out of 1,445 surfaces of the left caudate (failure rate being 1.31%), 15 out of 1,445 surfaces of the right caudate (failure rate being 1.04%), 7 out of 1,445 surfaces of the left hippocampus (failure rate being 0.48%), and 33 out of 1,445 surfaces of the right hippocampus (failure rate being 2.28%). We also note that the 19 left caudate surfaces with flaws were generated from the scans of 16 subjects while the 15 right caudate surfaces came from 9 subjects, the 7 left hippocampus surfaces came from 4 subjects, and the 33 right hippocampus surfaces came from 14 subjects. Such observations suggest that a failure for the proposed pipeline is more likely to recur in longitudinal scans of the same subject than on the dataset as a whole. In Figures 4, 5, we present the outputs in representative failure cases for the caudate (both left and right) and the hippocampus (both left and right) respectively.

In addition to qualitative assessment, we also conducted outlier analysis based on each surface's GL value. To be specific, outliers were defined as those whose GL values were outside the range[*Q*_1_−1.5(*Q*_3_−*Q*_1_), *Q*_1_+1.5(*Q*_3_−*Q*_1_)], where *Q*_1_ and *Q*_3_ respectively denote the 25 percentile and the 75 percentile of all structure-specific GL values. From this outlier analysis, we detected 15 outliers for the left caudate, 9 outliers for the right caudate, 6 outliers for the left hippocampus, and 26 outliers for the right hippocampus. These numbers agree well with our qualitative assessment results.

### The second experiment

The mean values and standard deviations of GL for the 12 subcortical surfaces, delivered by FSL-FIRST, are also listed in Table 5, from which we observed a similar level of smoothness as results from the proposed pipeline, both being significantly smoother than those from the gold standard and MALF. Comparing between the proposed pipeline and FSL-FIRST, the bilateral amygdalar surfaces from the proposed pipeline are much smoother than those from FSL-FIRST whereas an opposite pattern was observed for the bilateral hippocampal surfaces. Overall, those two methods have similar performance in terms of surface smoothness. With regards to the segmentation accuracy, as quantified by the DSCs (Table 6), the AVDs (Table 7), and PCCs (Table 8), the proposed pipeline significantly outperformed FSL-FIRST.

**Table 6:** The average Dice overlap coefficients between the gold standard and segmentations from the proposed method as well as those between the gold standard and FSL-FIRST over the 16 MRI scans of the first group for the 12 subcortical structures alongside the corresponding *p*-values obtained from Student's *t*-tests.

	**Proposed method**	**FSL-FIRST**	***p*-value**
Left caudate	0.913 ± 0.039	0.832 ± 0.025	3.548E-06
Right caudate	0.899 ± 0.027	0.834 ± 0.016	2.727E-08
Left pallidum	0.899 ± 0.024	0.818 ± 0.041	1.658E-05
Right pallidum	0.906 ± 0.022	0.797 ± 0.042	7.068E-08
Left putamen	0.928 ± 0.009	0.881 ± 0.023	1.314E-06
Right putamen	0.934 ± 0.009	0.882 ± 0.021	1.286E-09
Right thalamus	0.924 ± 0.011	0.898 ± 0.023	2.849E-04
Left thalamus	0.929 ± 0.008	0.902 ± 0.019	2.397E-03
Left amygdala	0.874 ± 0.020	0.779 ± 0.041	3.057E-07
Right amygdala	0.870 ± 0.025	0.776 ± 0.032	2.818E-07
Left hippocampus	0.909 ± 0.011	0.826 ± 0.024	6.721E-09
Right hippocampus	0.907 ± 0.014	0.833 ± 0.019	9.484E-10

**Table 7:** The average absolute volume differences between the gold standard and segmentations from the proposed method as well as those between the gold standard and FSL-FIRST over the 16 MRI scans of the first group for the 12 subcortical structures alongside the corresponding *p*-values obtained from Student's *t*-tests.

	**Proposed method**	**FSL-FIRST**	***p*-value**
Left caudate	0.078 ± 0.088	0.135 ± 0.042	5.273E-02
Right caudate	0.100 ± 0.079	0.099 ± 0.049	9.674E-01
Left pallidum	0.108 ± 0.079	0.179 ± 0.081	4.673E-02
Right pallidum	0.086 ± 0.071	0.244 ± 0.055	1.359E-05
Left putamen	0.035 ± 0.027	0.183 ± 0.059	5.703E-09
Right putamen	0.055 ± 0.023	0.147 ± 0.055	1.153E-06
Right thalamus	0.092 ± 0.039	0.083 ± 0.066	7.772E-01
Left thalamus	0.075 ± 0.036	0.061 ± 0.062	1.786E-01
Left amygdala	0.077 ± 0.045	0.132 ± 0.079	4.229E-02
Right amygdala	0.065 ± 0.072	0.205 ± 0.140	1.486E-03
Left hippocampus	0.076 ± 0.029	0.259 ± 0.087	7.400E-07
Right hippocampus	0.074 ± 0.033	0.188 ± 0.083	2.403E-04

**Table 8:** The Pearson product-moment correlation coefficients between the gold standard and segmentations from the proposed method as well as those between the gold standard and FSL-FIRST over the 16 MRI scans of the first group for the 12 subcortical structures alongside the corresponding *p*-values indicating the significance level of each correlation.

	**Proposed method**		**FSL-FIRST**	
	**PCC**	***p*-value**	**PCC**	***p*-value**
Left caudate	0.827	7.730E-05	0.953	1.129E-08
Right caudate	0.829	7.075E-05	0.948	2.528E-08
Left pallidum	0.741	1.031E-03	0.831	6.723E-05
Right pallidum	0.835	5.635E-05	0.921	4.022E-07
Left putamen	0.980	3.128E-11	0.946	3.024E-08
Right putamen	0.966	1.349E-09	0.965	1.475E-09
Right thalamus	0.908	1.160E-06	0.730	1.329E-03
Left thalamus	0.898	2.347E-06	0.706	2.237E-03
Left amygdala	0.641	7.484E-03	0.440	8.847E-02
Right amygdala	0.711	2.009E-03	0.097	7.217E-01
Left hippocampus	0.809	1.454E-04	0.568	2.179E-02
Right hippocampus	0.819	1.032E-04	0.576	1.962E-02

## Discussion

In this paper, we have developed a fully-automated shape generation pipeline for subcortical and ventricular structures of the human brain which preserves smoothness and anatomical topology in the surfaces. The performance of the pipeline has been validated on three datasets, both quantitatively and qualitatively. We found that, without sacrificing the accuracy, the resultant surfaces have high smoothness and correct anatomical topology. Based on visual examinations and outlier analyses on a large number of surfaces (1,445 in total for each structure), the pipeline has a very low rate of failure; to be specific, the failure rate is 0% for the putamen, the globus pallidus, the amygdala, the thalamus, and the lateral ventricle in both hemispheres, 1.31% for the left caudate, 1.04% for the right caudate, 0.48% for the left hippocampus, and 2.28% for the right hippocampus. As is exemplified in Figures 4, 5, the main cause of failure for the caudate and the hippocampus is segmentation inaccuracy incurred in the MALF based automated segmentation. Those two structures are both adjacent to the cerebrospinal fluid and it has been found that this makes them more susceptible to inaccuracy (Tang et al., 2013). Even for those two structures, the failure rates on the first and the third datasets are 0% while those on the second dataset are < 3% and we consider such results to be a strong indicator of the pipeline's capacity for high performance.

There are three main components in the pipeline: automated structure segmentation; creation of study-specific template shapes; and LDDMM-based shape filtering. For automated structure segmentation, we utilized a well-developed algorithm of our own group's creation, the diffeomorphic multi-atlas likelihood fusion. Using the first and the third datasets, which have the manual segmentations available, we again validated the performance of the MALF algorithm in terms of the automated segmentation of subcortical and ventricular structures. For this component, we can also use other automated structure segmentation algorithms, as long as the accuracy is sufficient, such as FreeSurfer (Fischl et al., 2002) and FSL-FIRST (Patenaude et al., 2011). FreeSurfer based segmentations have also been used for surface generation in existing works (Qiu and Miller, 2008; Qiu et al., 2009b; Tang et al., 2014). In FSL-FIRST, the segmentation of a subcortical structure of interest is actually obtained from its corresponding smooth surface. In other words, FSL-FIRST outputs both smooth surfaces and segmentations for subcortical structures. In that sense, it may be redundant to perform another round of surface generation based on segmentations from FSL-FIRST.

In this work, we did not compare the surface results from the proposed pipeline with those obtained from replacing our segmentation module with another one since that is essentially a comparison of various segmentation algorithms, which is not the goal of this paper. With that being said, we did validate the segmentation accuracy of our pipeline using the gold standard of the first dataset, with the DSCs ranging between 0.87 and 0.93 (Table 2), the AVDs ranging between 0.04 and 0.1 (Table 3), and the PCCs ranging between 0.72 and 1 (Table 4), as well as the third dataset (see Table S1 in the Supplementary Material 2).

For the second step, the creation of study-specific template shapes, we applied the Delaunay algorithm (Lee and Schachter, 1980; Shewchuk, 2002) for triangulating a carefully-selected manual segmentation for each structure of interest. The reason for using a manually created segmentation is 2-fold: firstly, a manual segmentation can guarantee correct anatomy and smoothness to some degree; secondly, we had previously generated the manual segmentations to serve as atlases in our automated structure segmentation phase, meaning no additional effort was required here. With that being said, we can also create a template shape based on an automated segmentation with sufficient accuracy, correct anatomy, and sufficient smoothness. The Delaunay algorithm is superior to the marching cubes algorithm in terms of smoothness of the resultant surfaces though it can fail in some cases, especially when the segmentation is flawed. Therefore, in this case, we were well-placed to generate the template shapes using the Delaunay algorithm since we could pay special attention to those surfaces. Meanwhile the marching cubes algorithm was better suited for the target segmentations.

In practice, there are two guiding rules in selecting the template surface: (1) the same definitions should be used in the automated segmentations of the target MRIs as in the segmentation of the template surface. For example, in this work, all automated segmentations of the first two datasets were obtained by using the atlases of the 16 subjects while the template surface was also obtained from this 16-subject pool. It may be inappropriate to use a template surface from a MALF-based segmentation definition to smooth an automated segmentation from FSL-FIRST; (2) It is better to select a template surface from the same study sample. In other words, it may be inappropriate to use a template surface from our HD study to smooth an automated segmentation from another study.

For the third step, LDDMM-based shape filtering, the key idea is to use a diffeomorphic transformation that can accurately deform the template shape to be very close to the target one while preserving the smoothness and topology of the template shape. LDDMM-surface is a validated algorithm that has been shown to yield sophisticated diffeomorphisms that can accurately register a pair of surfaces (Vaillant and Glaunès, 2005). According to our experiments on all three datasets, the deformed results, based on LDDMM-surface matching, are very close to the raw data (the target segmentations for which we aim to create their corresponding surfaces) while preserving the topology and smoothness of the template shapes. The high fidelity of the resulting surfaces to the target segmentations is somewhat of a double-edge sword; on the one hand, it guarantees high accuracy while on the other, it causes sensitivity to the inaccuracy induced in the segmentation process. In other words, when the segmentations are noisy (like those from the second dataset that the pipeline failed on), the resulting surfaces will inherit the noise (inaccuracy) of the segmentations from MALF. A potential solution is to utilize a much more robust variant of the LDDMM-surface matching, such as the one proposed by Tward and colleagues (Tward et al., 2016). Investigation of more advanced surface matching algorithms that are capable of maintaining a high fidelity to the segmentation while being robust to noisy subregions of the segmentations will be one of our future efforts. Furthermore, there are wholly separate registration approaches that can be applied to deforming surfaces, such as the 14 methods compared in (Klein et al., 2009). We did not compare here the surfaces generated by using different surface deformation approaches as that goes beyond the scope of this paper; to formulate the proposed pipeline.

This work was strongly motivated by the ongoing search for simpler, more effective, and more flexible pipelines capable of generating subcortical and ventricular surfaces with high smoothness and correct anatomy. According to our comparison results with another popular pipeline that directly outputs binary segmentations and smooth triangulated surfaces, namely FSL-FIRST, the surface results from the proposed pipeline have a similar degree of smoothness as those from FSL-FIRST, whereas the proposed pipeline's segmentation accuracy is significantly higher than FSL-FIRST for almost each of the 12 subcortical structures, which agrees with our previous findings (Tang et al., 2015c). This again may suggest a superiority of the proposed pipeline, although we must be aware of the potential unfairness given that a specific structure's definition may differ significantly for atlases used in MALF and those in FSL-FIRST. Compared with existing pipelines, the main contribution of this work, aside from the pipeline performance, is to have provided a general framework that can be easily adopted or modified according to one's own purpose; for example, to replace MALF with another segmentation algorithm that one favors more or to choose a template surface that one considers to be more suitable for a specific study.

One potential limitation of the proposed pipeline is that it is difficult to be sure that no subtle disease-related features were lost during this surface generation process. A way to partially address this question is to compare the disease-related features (via group comparison to a control group) obtained by using a set of surfaces created manually (to ensure accuracy) and those obtained by using a set of surfaces created from the proposed pipeline. However, given the lack of such a set of manually created surfaces involving both control and disease subjects, it is not possible to conduct such an experiment at this moment. We anticipate that as one of our future endeavors.

The statistical shape analysis of subcortical and ventricular structures of the human brain has become a topic of most considerable interest in contemporary research (Styner et al., 2003; Qiu and Miller, 2008; Qiu et al., 2010). We are confident that the proposed pipeline will further the development of this research field, especially in the investigations of HD.

## Author contributions

XT and MM: Contributed to the design of the entire pipeline; YL, ZC, and NH: Contributed to the analysis and evaluation experiments; HJ and JP: Contributed to the data acquisition; XT: Wrote the paper. All authors revised the manuscript critically for important intellectual content.

### Conflict of interest statement

MM owns an equal share in Anatomyworks LLC. The terms of this arrangement have been reviewed and approved by the Johns Hopkins University, in accordance with it conflict of interest policy. The other authors declare that the research was conducted in the absence of any commercial or financial relationships that could be construed as a potential conflict of interest.
