# Supplementary material for: A Fully-Automated Subcortical and Ventricular Shape Generation Pipeline Preserving Smoothness and Anatomical Topology
Source: Front Neurosci. 2018 May 17;12:321. doi: 10.3389/fnins.2018.00321 (PMC5966575; doi:10.3389/fnins.2018.00321)
Supplement: Supplementary file 1 [file Data_Sheet_1.DOCX]

**Table S1.** The average Dice overlap coefficients, the average absolute volume differences, as well as the Pearson product-moment correlation coefficients between every pairing of the three sets of segmentation results (manual segmentation, raw automated segmentation, and filtered automated segmentation) over the 20 testing MRI scans of the third dataset for each of the 12 subcortical structures.

|  | Manual VS. Raw Auto | | | Raw Auto VS. Filtered Auto | | | Manual VS. Filtered Auto | | |
| --- | --- | --- | --- | --- | --- | --- | --- | --- | --- |
|  | DSC | AVD | PCC | DSC | AVD | PCC | DSC | AVD | PCC |
| Left Caudate | 0.853 ± 0.072 | 0.120 ± 0.048 | 0.229 | 0.943 ± 0.010 | 0.017 ± 0.009 | 0.998 | 0.851 ± 0.069 | 0.112 ± 0.046 | 0.234 |
| Right Caudate | 0.853 ± 0.063 | 0.126 ± 0.044 | 0.516 | 0.942 ± 0.012 | 0.018 ± 0.009 | 0.999 | 0.853 ± 0.058 | 0.113 ± 0.041 | 0.536 |
| Left Pallidum | 0.864 ± 0.028 | 0.079 ± 0.059 | 0.475 | 0.948 ± 0.007 | 0.014 ± 0.006 | 0.998 | 0.862 ± 0.025 | 0.081 ± 0.055 | 0.491 |
| Right Pallidum | 0.863 ± 0.047 | 0.097 ± 0.104 | 0.803 | 0.948 ± 0.016 | 0.013 ± 0.006 | 0.999 | 0.862 ± 0.045 | 0.093 ± 0.098 | 0.804 |
| Left Putamen | 0.904 ± 0.027 | 0.051 ± 0.031 | 0.932 | 0.958 ± 0.012 | 0.007 ± 0.003 | 1.000 | 0.900 ± 0.026 | 0.046 ± 0.030 | 0.933 |
| Right Putamen | 0.904± 0.029 | 0.042 ± 0.029 | 0.931 | 0.958 ± 0.009 | 0.007 ± 0.004 | 1.000 | 0.900 ± 0.027 | 0.041 ± 0.029 | 0.928 |
| Right Thalamus | 0.903 ± 0.020 | 0.075 ± 0.045 | 0.949 | 0.966 ± 0.006 | 0.006 ± 0.003 | 1.000 | 0.905 ± 0.020 | 0.080 ± 0.048 | 0.946 |
| Left Thalamus | 0.905 ± 0.019 | 0.063 ± 0.039 | 0.955 | 0.967 ± 0.007 | 0.004 ± 0.004 | 1.000 | 0.906 ± 0.018 | 0.067 ± 0.041 | 0.952 |
| Left Amygdala | 0.785 ± 0.039 | 0.151± 0.026 | 0.435 | 0.934 ± 0.009 | 0.020 ± 0.006 | 0.999 | 0.787 ± 0.037 | 0.146 ± 0.025 | 0.442 |
| Right Amygdala | 0.781 ± 0.049 | 0.217 ± 0.033 | 0.426 | 0.932 ± 0.006 | 0.017 ± 0.007 | 0.999 | 0.782 ± 0.047 | 0.209 ± 0.020 | 0.435 |
| Left Hippocampus | 0.849 ± 0.025 | 0.065 ± 0.045 | 0.890 | 0.940 ± 0.004 | 0.013 ± 0.004 | 0.999 | 0.850 ± 0.025 | 0.061 ± 0.039 | 0.889 |
| Right Hippocampus | 0.854 ± 0.024 | 0.057 ± 0.026 | 0.880 | 0.941 ± 0.006 | 0.014 ± 0.004 | 0.999 | 0.851 ± 0.024 | 0.058 ± 0.030 | 0.882 |

**Table S2.** Smoothness quantification, as measured by the Geometric Laplacian, of the three sets of surface results (manual, raw automated (MALF), and filtered automated (proposed)) over the 20 testing MRI scans of the third dataset for the 12 subcortical structures.

|  | Manual | MALF | Proposed |
| --- | --- | --- | --- |
| Left Caudate | 635.644 ± 61.962 | 713.784 ± 143.734 | 198.335 ± 17.255 |
| Right Caudate | 632.816 ± 61.143 | 715.594 ± 151.768 | 222.229 ± 17.749 |
| Left Pallidum | 344.031 ± 24.198 | 297.035 ± 19.314 | 93.622 ± 2.649 |
| Right Pallidum | 336.267 ± 34.924 | 303.141 ± 21.812 | 85.892 ± 2.517 |
| Left Putamen | 780.236 ± 54.672 | 721.114 ± 51.029 | 189.854 ± 4.693 |
| Right Putamen | 771.567 ± 56.154 | 710.119 ± 58.989 | 194.823 ± 6.874 |
| Right Thalamus | 894.334 ± 92.431 | 822.640 ± 72.443 | 208.308 ± 6.389 |
| Left Thalamus | 937.735 ± 87.882 | 841.447 ± 73.305 | 214.505 ± 6.252 |
| Left Amygdala | 237.176 ± 28.443 | 250.816 ± 26.220 | 83.846 ± 3.154 |
| Right Amygdala | 244.339 ± 33.704 | 261.833 ± 21.120 | 81.399 ± 3.095 |
| Left Hippocampus | 746.966 ± 80.834 | 709.129 ± 61.270 | 201.074 ± 7.663 |
| Right Hippocampus | 803.860 ± 70.394 | 740.444 ± 66.311 | 201.816 ± 7.970 |
